# Conversion of CO_2_ into organic acids by engineered autotrophic yeast

**DOI:** 10.1073/pnas.2211827119

**Published:** 2022-11-16

**Authors:** Michael Baumschabl, Özge Ata, Bernd M. Mitic, Lisa Lutz, Thomas Gassler, Christina Troyer, Stephan Hann, Diethard Mattanovich

**Affiliations:** ^a^Austrian Centre of Industrial Biotechnology (ACIB), Vienna, 1190, Austria;; ^b^Department of Biotechnology, Institute of Microbiology and Microbial Biotechnology, University of Natural Resources and Life Sciences (BOKU), Vienna, 1190, Austria;; ^c^Department of Chemistry, Institute of Analytical Chemistry, University of Natural Resources and Life Sciences (BOKU), Vienna, 1190, Austria;; ^d^Present address: Institute of Microbiology, ETH Zurich, Zurich, 8093, Switzerland

**Keywords:** synthetic biology, metabolic engineering, carbon capture, organic acids, yeast

## Abstract

The increase of CO_2_ emissions due to human activity is one of the preeminent reasons for the present climate crisis. In addition, considering the increasing demand for renewable resources, the upcycling of CO_2_ as a feedstock gains an extensive importance to establish CO_2_-neutral or CO_2_-negative industrial processes independent of agricultural resources. Here we assess whether synthetic autotrophic *Komagataella phaffii* (*Pichia pastoris*) can be used as a platform for value-added chemicals using CO_2_ as a feedstock by integrating the heterologous genes for lactic and itaconic acid synthesis. ^13^C labeling experiments proved that the resulting strains are able to produce organic acids via the assimilation of CO_2_ as a sole carbon source. Further engineering attempts to prevent the lactic acid consumption increased the titers to 600 mg L^−1^, while balancing the expression of key genes and modifying screening conditions led to 2 g L^−1^ itaconic acid. Bioreactor cultivations suggest that a fine-tuning on CO_2_ uptake and oxygen demand of the cells is essential to reach a higher productivity. We believe that through further metabolic and process engineering, the resulting engineered strain can become a promising host for the production of value-added bulk chemicals by microbial assimilation of CO_2_, to support sustainability of industrial bioprocesses.

## Significance

Industrial biotechnology bears great potential to reduce CO_2_ emissions by producing chemicals from renewable agricultural feedstocks, however at the risk to compete with food production. While most industrially relevant organisms are heterotrophs there has been recent progress to equip some with a CO_2_ fixation pathway leading to autotrophic growth. We have recently developed a synthetic autotrophic strain of the industrial yeast *Komagataella phaffii*. Here we integrated the pathways to lactic and itaconic acid (two chemical building blocks) into this strain. Up to 2 g L^−1^ of itaconic acid were produced from CO_2_ as the only carbon source. This work paves the way toward net CO_2_ capturing into long living chemical products based on a synthetic autotrophic chassis strain.

Between 2011 and 2020 the annual average CO_2_ emission due to human activity exceeded 38 gigatons, of which around 22 gigatons are removed again from the atmosphere to terrestrial and ocean CO_2_ sinks. This imbalance, mostly due to combustion of fossil fuels, causes the steady increase in CO_2_ levels in the atmosphere which is one of the primary reasons for the climate crisis our planet is facing today (1).

Biologically produced fuels and commodity chemicals bear the potential to counteract this deleterious development, but the most common feedstocks used for bioproduction, such as glucose, sucrose and starch rely on agricultural production and are bearing the risk to threaten food security. Using autotrophic microorganisms as production platforms exploits the potential of CO_2_ itself as an alternative carbon source. In nature, autotrophic microorganisms play a major role in CO_2_ fixation by fixing 200 gigatons of CO_2_ every year (2). However, the rates of most natural microbial CO_2_ fixing pathways are low: the photosynthetic efficiency in cyanobacteria is limited to 1 to 2% which makes industrial processes using photoautotrophs economically less feasible (3). Chemoautotrophs may overcome this barrier as their energy harvesting processes are more efficient than light harvesting of photoautotrophs.

One well-known autotroph that is being developed for biological production of materials is *Cupriavidus necator* (formerly known as *Ralstonia eutropha*). By harvesting energy with a controlled Knallgas reaction these bacteria assimilate CO_2_ via the Calvin-Benson-Bessham (CBB) cycle. Besides naturally produced polyhydroxyalkanoates (PHA), metabolic pathway engineering enabled the production of several other chemicals (4–7).

Several chemolithotrophic bacteria were demonstrated as production hosts for various chemicals such as ethanol, 2,3-butanediol, butanol, isopropanol, acetone, or isobutyric acid via natural carbon assimilation pathways (8–11). In addition to natural CO_2_ fixing microorganisms, implementation of heterologous CO_2_ fixing pathways to heterotrophic microorganisms like *Myceliophthora thermophila* provided a mixotrophic strain that is able to produce malic acid with a higher yield compared to the parent strain in which only the reductive tricarboxylic acid (TCA) cycle is used for the production (12).

Natural chemoautotrophs have a large potential to convert CO_2_ to chemicals. However, they are often recalcitrant to genetic editing, have complex nutrient demands, or may require complex process technological solutions like the transfer of gaseous substrates and energy sources. To circumvent some of these limitations well established prokaryotic and eukaryotic production hosts have been engineered to assimilate CO_2_. The bacterial workhorse *Escherichia coli* and the yeast *Komagataella phaffii* (*Pichia pastoris*) were provided with the CBB cycle, enabling them to assimilate CO_2_ by using formate or methanol, respectively, as energy sources. Both engineered microorganisms can grow sustainably with CO_2_ as carbon source (13, 14). Conceptually formate and methanol are regarded as sustainable feedstocks for biotechnology when they are derived from CO_2_ by hydrogenation or electrochemical reduction (15).

To make an impact on the global CO_2_ household such autotrophic processes need to convert CO_2_ into bulk products. Besides ethanol, short chain organic acids are the second largest group of chemicals manufactured by industrial biotechnology. The market of biologically produced organic acids is expected to reach more than $36 billion by 2026 (16). The annual bioproduction of some of the key organic acids (citric, acetic, lactic, succinic acid) is more than 12 million metric tons (17–20). Recently, itaconic acid has also gained attention as a promising chemical building block with an estimated market increase to 170 kilotons per year and $260 million in 2025 (21). Lactic and itaconic acid are feedstocks for polymer production so that they, and other biobased commodity chemicals compete for the annual polymer production of more than 300 million tons, a volume that denotes a significant impact on the global CO_2_ balance.

**Fig. 1.**
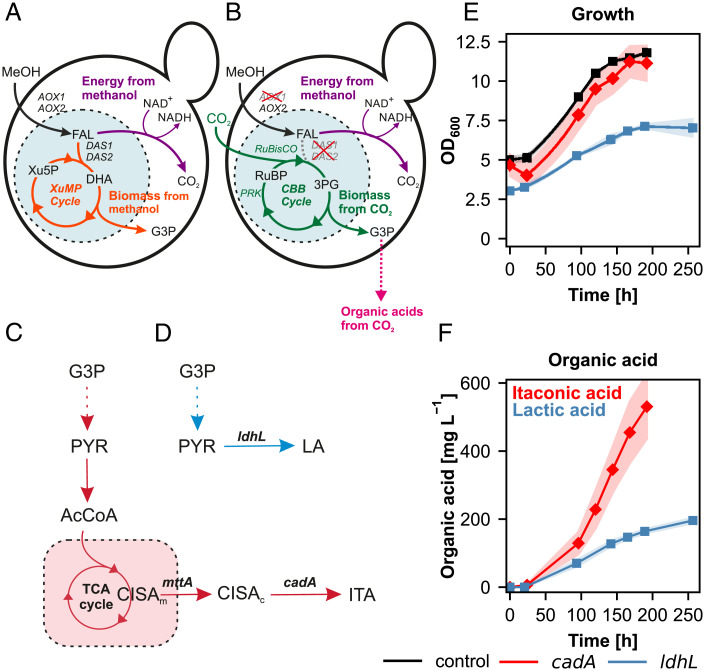
Expression of *cadA* and *ldhL* enables organic acid production in synthetic autotrophic *K. phaffii*. (*A–D*) Schematic pathways. (*A*) In wild-type *K. phaffii* methanol is oxidized to formaldehyde (black arrow) and assimilated in the XuMP cycle (orange arrows) or dissimilated to CO_2_, respectively (purple arrow). (*B*) synthetic autotrophy in *K. phaffii*: the native assimilatory branch of methanol utilization was interrupted by deleting *DAS1* and *DAS2* (dashed gray line). *AOX1* was knocked out to reduce the rate of formaldehyde formation which could be toxic to the cells. RuBisCO and *PRK* were integrated to complete a functional CBB cycle (green arrows). Additionally, two bacterial chaperones, *groEL* and *groES*, were overexpressed to assist the folding of RuBisCO. *TDH3*, *PGK1*, *TKL1*, *TPI1* carrying each a peroxisomal targeting signal were overexpressed to assure the localization of the entire CBB cycle in peroxisomes. More details about the engineering strategy can be found in ref. (13). (*C*) Itaconic acid (red) and (*D*) lactic acid production (blue), (*E*) growth profiles, and (*F*) organic acid production profiles of the producing strains and the control. Time axis corresponds to the production phase under autotrophic conditions. At least three biological replicates were used in the screening to monitor the producing strains. Shades represent the SDs (±). 3PG: 3-phosphoglycerate, AcCoA: acetyl-coenzyme A, *AOX1* and *AOX2*: alcohol oxidase 1 and 2, *cadA*: cis-aconitate decarboxylase, CBB cycle: Calvin-Benson-Bassham cycle, CISA_c_: cytosolic cis-aconitate, CISA_m_: mitochondrial cis-aconitate, *DAS1* and *DAS2*: dihydroxyacetone synthase 1 and 2, DHA: dihydroxyacetone, FAL: formaldehyde, G3P: glyceraldehyde 3-phosphate, ITA: itaconic acid, LA: lactic acid, *ldhL*: L-lactate dehydrogenase, MeOH: methanol, *mttA*: mitochondrial tricarboxylic acid transporter, NAD^+^/NADH: nicotinamide adenine dinucleotide, *PRK*: phosphoribulokinase, PYR: pyruvate, RuBP: ribulose 1,5-bisphosphate, *RuBisCO*: ribulose 1,5-bisphosphate carboxylase/oxygenase, Xu5P: xylulose 5-phosphate, XuMP cycle: xylulose monophosphate cycle.

Here, we set out to evaluate if the autotrophic *K. phaffii* strain can be used as a platform for organic acid production. Synthetic autotrophy was introduced to *K. phaffii* by converting the native peroxisomal methanol assimilation pathway, the xylulose monophosphate (XuMP) cycle, into the CBB cycle (13). To achieve that, the formaldehyde assimilating enzyme dihydroxyacetone synthase (DAS) was replaced by a bacterial ribulose 1,5-bisphosphate carboxylase/oxygenase (RuBisCO), and phosphoribulose kinase (PRK) from spinach was added to supply ribulose bisphosphate from XuMP precursors. Four yeast glycolytic enzymes were targeted to peroxisomes to close the CBB cycle to glyceraldehyde 3-phosphate (G3P), both as an intermediate and the product of the assimilation cycle (Fig. 1 *A* and *B*). In the present work, using modular synthetic biology tools, we implemented the genes for lactic and itaconic acid synthesis plus accessory genes into the *K. phaffii* genome and demonstrate that the autotrophic *K. phaffii* strain is capable of producing organic acids solely from CO_2_ as carbon source.

## Results

### Production of Organic Acids in Synthetic Autotrophic *K. phaffii*.

Itaconic acid is produced through the decarboxylation of cis-aconitate into itaconic acid by cis-aconitate decarboxylase encoded by *cadA* in *Aspergillus terreus* (Fig. 1*C*). The *cadA* gene was expressed under a strong methanol inducible promoter (P*_AOX1_*) coupling product formation to CO_2_ assimilation via the synthetic CBB cycle which is controlled by methanol-induced promoters (13). As autotrophic growth of the strains is slow we used a two-phase screening protocol, first growing the cells on glycerol for biomass formation for 22 to 24 h. In the second phase, cells were transferred to the production medium with an initial OD_600_ of 3 to 4 where air is enriched with 5% CO_2_ as the only carbon source, and 1% methanol is added for production of NADH and ATP. Calculated specific growth and production rates and yields of the screened strains are given in *SI Appendix*, Table S1. We screened the producing clones for 200 h under autotrophic conditions in comparison to the control strain without *cadA* (Fig. 1*E*). Expression of *cadA* did not affect growth, and the specific growth rates through most of the cultivation period were about 0.007 h^−1^ while the control strain had a specific growth rate of 0.006 h^−1^. The expression of a single gene, *cadA*, was sufficient for the cells to produce itaconic acid up to 530 mg L^−1^ at the end of the production phase with a specific production rate of 2.3 mg g^−1^ h^−1^ (Fig. 1*F*). Using a faster growing synthetic autotrophic strain carrying a mutation in the PRK gene (22) did not improve the final titers, but even led to lower specific production rates (*SI Appendix*, Fig. S1). The control strain that lacks *cadA* did not produce any itaconic acid during the cultivation as wild-type *K. phaffii* does not harbor necessary genes for itaconic acid synthesis.

L-lactic acid is produced via the reduction of pyruvate to L-lactic acid catalyzed by L-lactate dehydrogenase encoded by *ldhL* in *Lactobacillus plantarum*. To enable lactic acid production in *K. phaffii* the *ldhL* gene was expressed under the control of the strong methanol inducible promoter of the *AOX1* gene (Fig. 1*D*). The resulting strain was able to produce up to 200 mg L^−1^ after 250 h of production time (Fig. 1*F*) with a specific production rate of 0.85 mg g^−1^ h^−1^. Growth was slightly reduced compared to the control strain with a specific growth rate of 0.005 h^−1^ (Fig. 1*E*). *K. phaffii* control strains that do not have a heterologous lactate dehydrogenase do not produce any detectable lactic acid.

### Balancing the Coexpression of *cadA* and *mttA* Improves Production of Itaconic Acid.

**Fig. 2.**
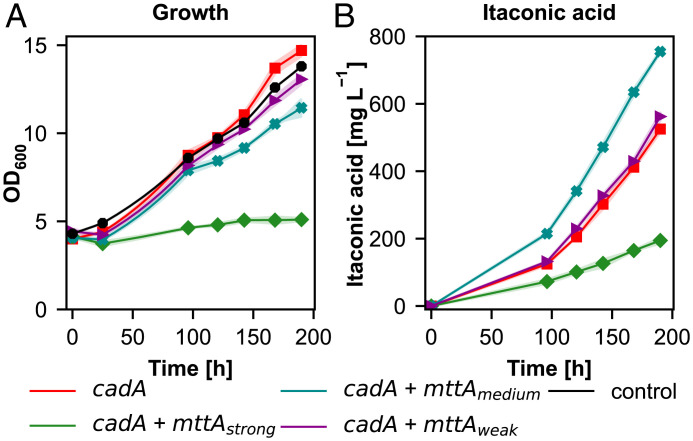
Fine-tuning the balance of expression of *cadA* and *mttA* increases itaconic acid production. (*A*) Growth profiles, (*B*) itaconic acid production profiles of the itaconic acid producing strain and the control. Time axis corresponds to the production phase under autotrophic conditions. At least three biological replicates were used in the screening to monitor the producing strains. Shades represent the SDs (±).

Itaconic acid metabolism requires two key genes: cis-aconitate decarboxylase (*cadA*) and a mitochondrial tricarboxylic acid transporter (*mttA*) to shuttle the substrate, cis-aconitate, to the cytosol where *cadA* is localized. Coexpression of *mttA* with *cadA* was found to increase the itaconic acid production titers in *Aspergillus niger* (23, 24). To investigate the effect of the coexpression of *mttA*, we used three different promoters with different strengths: P*_FDH1_* as a strong, P*_POR1_* as a medium and P*_PDC1_* as a weak promoter (25). We found that balancing the coexpression of *cadA* and *mttA* is crucial to increase itaconic acid productivity (Fig. 2). Using the strong *FDH1* promoter severely impaired growth and the final itaconic acid titers were only around 190 mg L^−1^ at the end of the cultivation. As the cells did not grow, a higher specific productivity is expected, but the final titer was 4.4-fold lower than with overexpression of *cadA* alone leading to 1.4-fold lower specific productivity (1.3 mg g^−1^ h^−1^). However, when we used a medium strength (P*_POR1_*) or a weak promoter (P*_PDC1_*), growth was nearly restored (almost 80% of the cadA strain was achieved) and the final titers were improved. Using a medium strength promoter resulted in the highest titer (approximately. 780 mg L^−1^) and highest specific productivity (3.2 mg g^−1^ h^−1^). Therefore, this strain was selected for further experiments.

### Deletion of *CYB2* Prevents Lactic Acid Consumption.

**Fig. 3.**
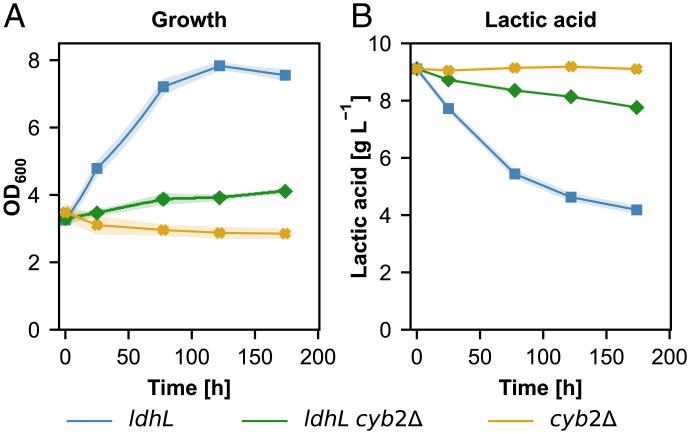
Deletion of *CYB2* prevents lactic acid consumption. (*A*) Growth profiles, (*B*) lactic acid consumption profiles of the lactic acid producing strain, the lactic acid producing *cyb2*Δ strain, and the *cyb2*Δ strain. Time axis corresponds to the cultivation time on media containing lactic acid under nonautotrophic conditions. At least three biological replicates were used in the screening to monitor the producing strains. Shades represent the SDs (±).

Under autotrophic conditions the cells face severe carbon limiting conditions. Since lactic acid can be efficiently converted back to pyruvate it could serve as a potential carbon source which would reduce the final lactic acid yield. Indeed, lactic acid consumption was seen in preliminary experiments (*SI Appendix*, Fig. S2). To overcome this bottleneck, the *CYB2* gene was knocked out in the lactic acid production strain. *CYB2* encodes for a L-lactate cytochrome *c* oxidoreductase located in the mitochondrial intermembrane space. Deletion of this gene abolished growth on L-lactate in *Saccharomyces cerevisiae* (26, 27). To evaluate the effect of the deletion in *K phaffii* we performed cultivations using lactate as the sole carbon source. The *ldhL* strain was able to grow on L-lactic acid with a maximum growth rate of around 0.01 h^−1^ (Fig. 3*A*). Lactate concentration was reduced from 8.5 g L^−1^ to 4.0 g L^−1^ throughout the cultivation of this strain (Fig. 3*B*) resulting in an average specific consumption rate of 28 mg g^−1^ h^−1^. A nonproducing control strain harboring the *CYB2* knockout (*cyb2*Δ*)* was not able to grow on L-lactate nor to consume lactic acid. The production strain harboring the *CYB2* knockout (*ldhL cyb2*Δ) could barely grow with a maximum growth rate of around 0.002 h^−1^. This strain was able to consume small amounts of lactic acid with a reduction in lactic acid concentration from 8.5 g L^−1^ to 7.2 g L^−1^. The specific lactic acid consumption rate was reduced nearly threefold compared to the nonknockout strain resulting in 11 mg g^−1^ h^−1^.

**Fig. 4.**
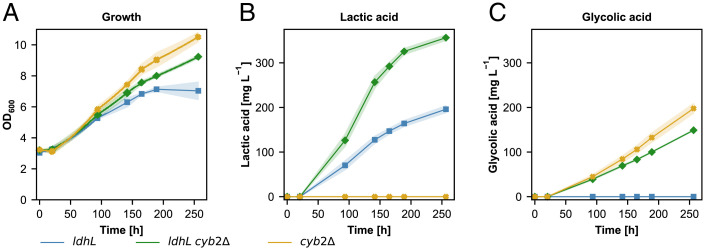
Deletion of *CYB2* improves lactic acid production in synthetic autotrophic *K. phaffii*. (*A*) Growth profiles, (*B*) lactic acid production profiles, (*C*) glycolic acid production profiles of the lactic acid producing strain and the control. Time axis corresponds to the production phase under autotrophic conditions. At least three biological replicates were used in the screening to monitor the producing strains. Shades represent the SDs (±).

In the next step, the capabilities of these strains to produce lactic acid were evaluated under autotrophic conditions. As expected, the control strain harboring the *CYB2* knockout did not produce any lactate (Fig. 4). The production strain harboring the *CYB2* knockout resulted in an increased lactic acid production reaching a final titer of around 350 mg L^−1^ with a specific production rate of 1.3 mg g^−1^ h^−1^. Growth of both knockout strains was slightly higher compared to the nonknockout strain with the nonproduction strain resulting in fastest growth (Fig. 4*A*). By transforming the genetic setup into a faster growing version of the synthetic autotrophic strain harboring a mutation in the *PRK* gene (22) we could not further enhance production of lactic acid. The nonknockout strain was only producing 50 mg L^−1^. In this strain background the knockout of *CYB2* could increase the titer up to fourfold reaching up to 200 mg L^−1^ (*SI Appendix*, Fig. S3).

Interestingly all strains harboring the *CYB2* knockout produced glycolic acid. Under autotrophic conditions the non-lactic acid producing knockout strain was able to produce up to 200 mg L^−1^ and the lactic acid strain accumulated 150 mg L^−1^ after 250 h of production phase (Fig. 4*C*).

### Reverse ^13^C Labeling Proves That Carbon in Lactic and Itaconic Acid is Incorporated From the Captured CO_2_.

**Fig. 5.**
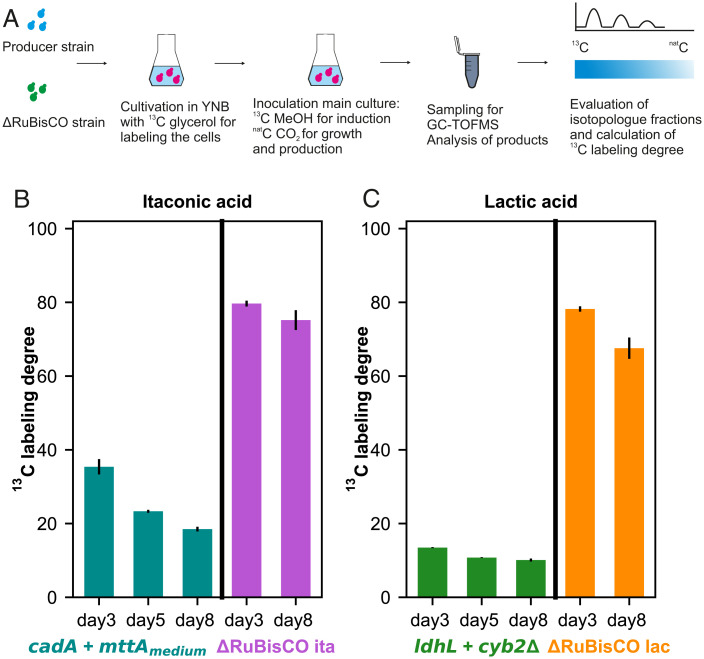
Reverse labeling confirms that carbon in lactic and itaconic acid is incorporated from the captured CO_2_. (*A*) Experimental design of the reverse labeling approach. After labeling the cells on ^13^C glycerol, we inoculated them into YNB and supplemented them with ^nat^C CO_2_ (CO_2_ with natural isotope distribution) and ^13^C MeOH. Samples were taken at different time points (day 3, day 5 and day 8) for the analysis with GC-TOFMS to monitor the change in the^13^C labeling pattern of the produced organic acids. (*B*) ^13^C labeling degree at different timepoints of the produced itaconic acid using the production strain on the left and the nongrower control. (*C*) ^13^C labeling degree at different timepoints of the produced lactic acid using the production strain on the left and the nongrower control. Error bars indicate the SD of 3 biological replicates. Detailed isotopologue distribution of the carbon atoms can be found in *SI Appendix*, Fig. S5.

After demonstrating that the CO_2_ capturing *K. phaffii* can produce organic acids, we wanted to prove that the carbon in the organic acids is incorporated from the captured CO_2_ via the CBB cycle. We used a reverse labeling approach (Fig. 5*A*). We first fully labeled the biomass by cultivating the strains for at least 10 generations on ^13^C glycerol as the only carbon source. The resulting ^13^C content measured using EA-IRMS was 97 ± 2% for the lactic acid and 94 ± 3% for the itaconic acid producing strains, respectively.

After ^13^C enrichment, ^nat^C CO_2_ (CO_2_ containing carbon with a natural isotope distribution of 1.1% ^13^C) was supplemented as the C source, and ^13^C MeOH was used for induction and energy harvest. As shown by Gassler et al. (13) ^12^C can only be derived from CO_2_ in this experimental setup with our strains. Therefore, we expect a drop in the ^13^C labeling degree of our samples. We took samples to monitor the transient changes of the isotopologue patterns and ^13^C labeling degree (according to the equation denoted in the *SI Appendix*, *Supplementary Methods*) of the products (Fig. 5 *B* and *C* and *SI Appendix*, Fig. S4) on day 3 (time where we start to measure significant amounts of organic acid), day 5 and day 8. In addition to the producing strain, we also used a RuBisCO knockout producer strain as a control that should not be able to fix CO_2_ due to the interrupted CBB cycle. Still these strains produced low amounts of itaconic or lactic acid, presumably from reserve metabolites or residual intracellular metabolite pools, however providing enough material to evaluate the isotopologue patterns of these products.

*SI Appendix*, Fig. S5 shows how the ^13^C labeling pattern of itaconic and lactic acid changed during the production. As expected, the ^12^C contents of both organic acids were increased throughout the cultivation. For itaconic acid, the ^13^C labeling degree decreased from 35% (day 3) to 18% (day 8) (Fig. 5*B*). The low amounts of itaconic acid produced by the delta RuBisCO producer strain (∼0.4 mmol L^−1^) resulted in a high ^13^C labeling degree of 75% at the end of the cultivation.

For lactic acid, the ^13^C labeling degree dropped to only 10% at the end of the cultivation. Lactic acid is derived from pyruvate which is much closer to the entry of the CBB cycle into the central carbon metabolism than cis-aconitate, the precursor for itaconic acid. Therefore, more of the unlabeled carbon coming from the CBB cycle will appear at early time points in lactic acid than in itaconic acid, thus a higher ^12^C content of lactic acid is to be expected in the transient ^12^C enrichment phase observed here. Additionally, the ^12^C pattern of the lactic acid produced by the delta RuBisCO producer strain differs significantly from the itaconic acid producer: The ^13^C labeling degree of the produced lactic acid (around 0.4 mmol L^−1^) was 68% at the end of the cultivation (Fig. 5*C*). Natural CO_2_ fixation pathways, like anaplerotic reactions, might have contributed to the incorporation of ^12^CO_2_ into the produced organic acids.

To calculate the expected ^13^C labeling degree, we built a metabolic model including CO_2_ capture, glycolysis, anaplerotic reaction from pyruvate to oxaloacetate and TCA cycle reactions, including itaconic or lactic acid metabolism genes and calculated the theoretical isotopologue distributions of the products using isotopomer network compartmental analysis [INCA; (28)] using the secretion rates of itaconic and lactic acid as constraints (*SI Appendix*, Table S2). Expected labeling degrees were calculated on day 8 as 23% for the itaconic and 18% for the lactic acid producer strains, respectively, which is equivalent to a 77% or 82% CO_2_ incorporation. Experimentally derived CO_2_ incorporation was 6% and 10% higher than predicted for itaconic and lactic acid, respectively, which may be due to the contribution of natural CO_2_ fixing reactions which were not included in the metabolic model. Additionally, the higher gap between the measured and predicted values for lactic acid producing strains could be due to the degradation rate of lactic acid which overlays the synthesis rate.

Complementary to the reverse labeling experiment we tested if the production depends on the supply of CO_2_. After switching the cells from 5 to ambient CO_2_ concentration production and growth was nearly stopped (*SI Appendix*, Fig. S6).

### Modification of Process Conditions Further Enhances the Production of Itaconic Acid but not of Lactic Acid.

**Fig. 6.**
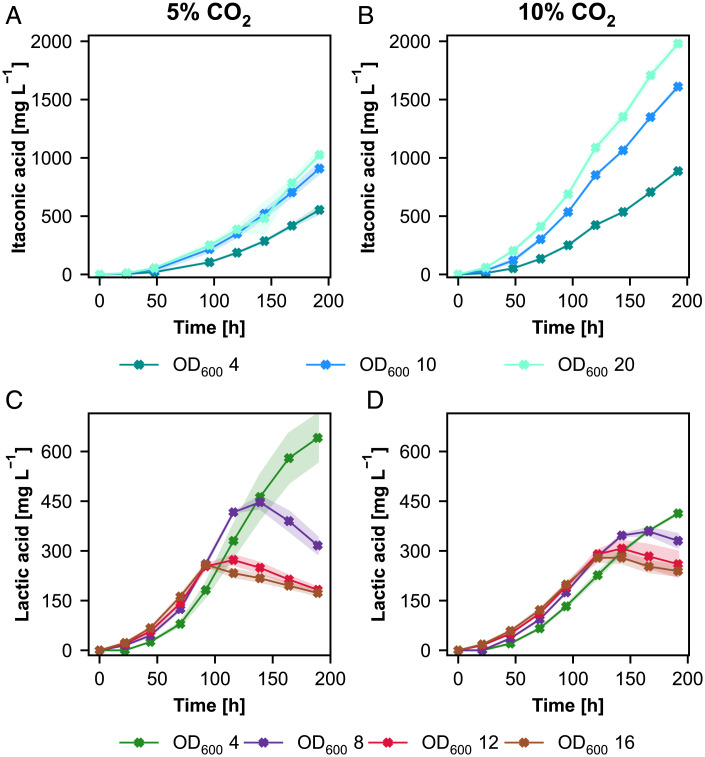
Modification of process conditions shows that each product has its own requirements for an optimum bioprocess. (*A*, *B*) Itaconic acid production profiles and (*C*, *D*) lactic acid production profiles at 5% CO_2_ and 10% CO_2_ supply, respectively, of producing clones and the control. Time axis corresponds to the production phase under autotrophic conditions. At least two biological replicates were used in the screening to monitor the producing strains. Shades represent the SDs (±).

We sought to modify the process conditions in the shake flask to see their effect on the production and screened the cells at 10% CO_2_ supply and with different initial OD_600_ values (between 4 and 20). For itaconic acid production, we observed a positive correlation between higher initial ODs and production at 5% CO_2_ supply. The higher the initial OD, the higher final titers were obtained, reaching more than 1 g L^−1^ (OD_600_ = 20) (Fig. 6*A*). However, specific growth and production rates decreased with increasing initial OD. Increasing the CO_2_ supply did not help to increase the specific growth rates which stayed also the same for all different initial ODs. However, a significant increase in the final titers was obtained (again in positive correlation with increasing ODs) when 10% CO_2_ was supplied. With a start OD_600_ = 20 at 10% CO_2_ almost 2 g L^−1^ itaconic acid (Fig. 6*B*) could be reached at the end of the production phase with a specific productivity of 2.7 mg g^−1^ h^−1^.

For lactic acid production, we obtained a different production profile. Increasing the initial OD did not increase the final titer. We screened different starting OD values in the range from OD_600_ = 4 to 16 (*SI Appendix*, Fig. S7*C*). In the beginning of the production phase higher OD led to slightly higher titers but later got outperformed by the lowest starting OD. Higher biomass cultures showed that lactic acid was consumed again toward the end of the cultivation, indicating an increase of substrate limitations with increased biomass. With the lowest starting OD, 600 mg L^−1^ lactic acid were produced, compared to 160 mg L^−1^ lactic acid for the highest starting OD (Fig. 6*C*). Interestingly, the glycolate production profile was different. OD_600_ = 8 to 16 resulted in very similar production profiles ending up with 170 mg L^−1^ glycolate. OD_600_ = 4 ended up with a slightly lower glycolate titer of 120 mg L^−1^ (*SI Appendix*, Fig. S7*E*). To overcome some possible limitations with higher starting ODs we doubled the CO_2_ concentration from 5 to 10%. Growth was very similar compared to our standard CO_2_ concentrations (*SI Appendix*, Fig. S7*D*). Increased CO_2_ supply was able to increase the production for cultures starting with OD_600_ = 12 and 16. OD_600_ = 8 ended up with similar titers compared to 5% but without the peak after 140 h of cultivation time. Interestingly OD_600 =_ 4 resulted in decreased lactic acid production with elevated CO_2_ concentrations (Fig. 6*D*). The glycolate production profile was similar but with a slight reduction in titers at 10% CO_2_ resulting in 100 mg L^−1^ for OD_600_ = 8 to 16 and 70 mg L^−1^ for OD_600_ = 4 respectively (*SI Appendix*, Fig. S7*F*).

### Bioreactor Cultivations: Upscaling Is Not Straightforward.

**Fig. 7.**
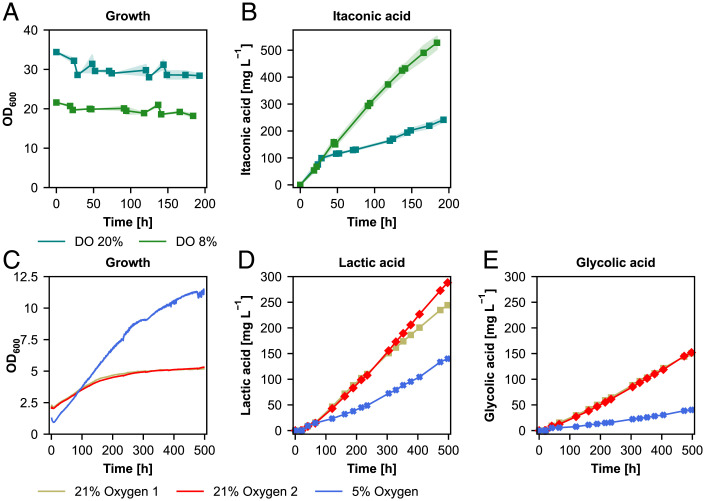
Bioreactor cultivations show each process should be designed based on the specific requirements of the target product. (*A*) Growth and (*B*) itaconic acid production profile at constant DO concentrations 8% and 20%. (*C*) Growth, (*D*) lactic, and (*E*) glycolic acid production profile at 21% and 5% inlet oxygen concentrations. Time axis corresponds to the production phase under autotrophic conditions. Bioreactor cultivations for itaconic acid production were done in duplicates and shades represent the SD (±). For lactic acid results of each fermentation run are shown separately.

Based on the screening results with different process conditions, we proceeded with bioreactor cultivations to investigate the production capacity of the producing strains at a larger scale. Depending on the initial screenings, we designed different bioreactor cultivation setups. For itaconic acid production, after a batch phase with glycerol to reach an OD_600_ of 20 to 30 (corresponding to 4 to 6 g L^−1^ dry cell weight (DCW)), cells were fed with 10% CO_2_ as the carbon source and feeding with 1% MeOH every 2 d. We tested two different dissolved oxygen (DO) concentrations: we either kept the DO above 20%, or at 8% by decreasing the oxygen concentration in the inlet gas. Similar to the screenings in shake flasks, the cells did not grow and the biomass remained at 4 to 6 g L^−1^ until the end of the cultivation. The DO concentration in the medium has an effect on the itaconic acid production: at lower DO concentration, higher itaconic acid concentrations could be achieved than at a higher DO. However, we could not achieve as high itaconic acid concentrations as we could reach in the shake flask: the highest titer was 530 mg L^−1^ with a specific productivity of 0.74 mg g^−1^ h^−1^ (Fig. 7*B*).

The initial screenings of different process conditions (Fig. 6 *C* and *D*) indicated that a low biomass concentration was better for lactic acid production. Therefore, we performed bioreactor cultivations starting with a low biomass concentration (OD_600_ = 2) and tested two different oxygen concentrations with 21% in the inlet air (uncontrolled DO concentration) and 5% in the inlet air (and keeping the DO concentration at 8%). Reducing the oxygen concentration led to nearly doubling of the growth rates of the lactic acid producing strains (Fig. 7*C*). Similar as for itaconic acid, the oxygen concentration also influenced the lactic acid production of the cells. In contrast to itaconic acid, high oxygen levels were beneficial for production. At ambient oxygen levels up to 260 mg L^−1^ were produced with a specific productivity of 0.76 mg g^−1^ h^−1^(Fig. 7*D*). Lowering the oxygen supply reduced the final titer to 130 mg L^−1^ and the specific productivity to 0.22 mg g^−1^ h^−1^. A similar pattern was observed for the production of glycolic acid in this strain. At high oxygen levels up to 150 mg L^−1^ and at low levels up to 40 mg L^−1^ were produced (Fig. 7*E*).

## Discussion

We have demonstrated previously that the yeast *K. phaffii* can be engineered to assimilate CO_2_ instead of methanol as its only carbon source (Fig. 1 *A* and *B*) (13). Here we show that this synthetic chemoautotrophic *K. phaffii* can be used as a platform to produce organic acids from CO_2_.

Expression of a cytosolic cis-aconitate decarboxylase from *A. terreus*, the key gene that encodes the enzyme for the decarboxylation reaction of cis-aconitate to itaconic acid, enabled *K. phaffii* to reach more than 500 mg L^−1^ itaconic acid final titers (Fig. 1*F*). Coexpression of a mitochondrial cis-aconitic acid transporter gene, *mttA*, was shown to be beneficial for itaconic acid production by creating a pull effect from mitochondria to the cytosol and increasing the availability of cis-aconitate for CadA (29, 30). Our results demonstrate that balancing the expression levels of *cadA* and *mttA* is essential to reach higher productivities (31) and that the strengths of the promoters used for expression of *cadA* and *mttA* do not have to be necessarily at the same level: using a weaker promoter for coexpression of *mttA* than the one used for *cadA* led to increased itaconic acid concentrations and specific productivity, reaching more than 700 mg L^−1^ final titers (Fig. 2).

In addition to itaconic acid, we also showed that lactic acid can be produced successfully with the synthetic autotrophic *K. phaffii* strain. Integration of a bacterial lactate dehydrogenase gene was sufficient to produce lactic acid titers of 200 mg L^−1^ (Fig. 1*F*). However, the produced lactic acid also served as a potential carbon source (Fig. 3) during the growth in the shake flask screenings as the cells were facing limited cultivation conditions while growing on CO_2_ as the sole C source. Therefore, our first engineering target was the reduction of lactic acid consumption. With a single knockout of *CYB2*, which encodes for a L-lactate cytochrome *c* oxidoreductase located in the mitochondrial intermembrane space, growth on lactate was nearly fully abolished. The specific lactic acid consumption rate decreased threefold in the final production strain expressing *ldhL* and harboring the *CYB2* knockout. Similar to *S. cerevisiae* Cyb2 (26) it is also the only enzyme responsible for lactic acid oxidation in wildtype *K. phaffii*. The small amounts of lactic acid consumption which was observed in the production strain could be explained by the heterologous lactate dehydrogenase performing the reverse reaction. The oxidation of lactate by LdhL is unfavorable compared to the reduction with a *K*_M_ for lactate more than 20× higher compared to pyruvate, nevertheless the results indicate that there is a certain degree of lactate oxidation catalyzed by the LdhL (32). The decrease in lactate oxidation in the *cyb2Δ* strain is probably the main reason for the increased lactic acid titers in our final production strain. An interesting aspect of the deletion of *CYB2* is the increased glycolic acid production in the synthetic autotrophic *K. phaffii* strain. This is probably linked to the oxygenation reaction of the RuBisCO enzyme. The products of this reaction are one molecule each of 3-phosphoglycerate and 2-phosphoglycolate, which can be afterward dephosphorylated to glycolate. This hypothesis correlates with a decrease in glycolic acid secretion when the CO_2_ concentration was increased (*SI Appendix*, Fig. S7) and oxygen concentration was reduced (Fig. 7*E*), conditions which should limit the oxygenation reaction of RuBisCO.

Reverse labeling experiments provided a further proof of the incorporation of captured CO_2_ into the organic acids produced. With ^nat^C CO_2_ as the carbon source we observed a decreasing ^13^C labeling degree over time. After 8 d, the ^13^C labeling degree had dropped to 18% and 10% for itaconic and lactic acid, respectively, indicating that the carbon is indeed derived from CO_2_ in the production phase. The faster reverse labeling obtained with lactic acid can be explained by being metabolically closely (and linearly) linked to the CBB cycle. For itaconic acid the intermediate TCA cycle pools first need to be enriched with label before complete enrichment of the product is obtained.

We also observed some incorporation of ^12^C atoms into the organic acids by the nongrowing strains. The ^13^C labeling degree had dropped to 75% for itaconic acid and 68% for lactic acid respectively at the end of the cultivation of the nongrowing strains. The unlabeled starting biomass (∼5%) might have caused an increase in the ^12^C content of the produced organic acids. In addition, natural CO_2_-fixing pathways might have also contributed to the incorporation of ^12^CO_2_ into the produced organic acids, thereby increasing the ^12^C content. One apparent pathway is the anaplerotic reaction, carboxylating pyruvate into oxaloacetate by the native pyruvate carboxylase. Another possible explanation for the itaconic acid producing strains could be the reverse TCA cycle. To assess the possibilities, we modeled the theoretical isotopologue distribution of the products using the metabolic model described above (*SI Appendix*, Table S2) where either forward or reverse TCA cycle reactions are active. However, we could not verify whether the reverse TCA cycle is active or not. The incorporation of CO_2_ through natural metabolic pathways still remains to be resolved.

*K. phaffii* is one of the suitable platforms for the production of recombinant proteins, enzymes or chemicals (33). To assess the feasibility to produce chemicals with the synthetic chemoautotrophic *K. phaffii*, we investigated also process conditions. Each product has its own requirements, and the process needs to be optimized accordingly. Increasing the initial biomass did not increase lactic acid titers, while starting with a higher biomass increased the final titers for itaconic acid (at a cost of lower specific production rates). One reason is that unlike itaconate, lactate can be used as a carbon source by *K. phaffii*. Even though the knockout of *CYB2* blocked the lactic acid assimilation, a small portion could be still consumed in strains expressing *ldhL* (Fig. 3). In contrast to lactic acid production, itaconic acid production was significantly improved when the CO_2_ concentration was increased from 5 to 10%, showing that the CO_2_ availability at high concentration is one of the key steps to increase productivity. This led to an almost 2-fold increase in the final titers reaching ∼2 g L^−1^ itaconic acid at the end of the process. However, considering a well-known bioprocess like citric acid production which yields more than 200 g L^−1^, the organic acid titers we reached in a shake flask are still far from a feasible industrial process (23). So far, the highest itaconic acid titers in bioreactors reached by a native producer and an engineered yeast is 160 and 55 g L^−1^, respectively (34, 35). We could produce up to 600 mg L^−1^ of lactic acid using our autotropic *K. phaffii* strain which is already close to the titers reached in cyanobacteria of ∼1 g L^−1^ (36, 37) but still far away from industrially relevant titers of far over 100 g L^−1^ (38).

However, upscaling the process to bioreactors was not straightforward. Using the best process conditions for organic acid production at different oxygen availability, we could not obtain as high titers as in the shake flask screenings. Nevertheless, it is apparent that a fine balancing between the carboxylation and oxygenation reaction of RuBisCO enzyme is crucial. Oxygen is required for the oxidation reaction of methanol to harvest energy and an important factor for the production of itaconic acid (29, 39), but also acts as a competitor for RuBisCO decreasing the CO_2_ fixing rates (40) which eventually leads to lower organic acid production. Therefore, maintaining the balance between the carbon dioxide and oxygen transfer rates is of great importance here to fine-tune the anabolic reactions and energy requirements of the cell to reduce by-product formation and reach higher productivities. One approach here could be replacing alcohol oxidase by a methanol dehydrogenase to decrease the oxygen demand and eliminate the oxygenation side reaction. Other process conditions including pH, temperature, and methanol feeding regime known to be affecting lactic or itaconic acid production or the production capacity of *K. phaffii* should also be optimized (41–44). Additionally, redirecting the flux toward target product through metabolic engineering coupled with a model-based approach (45) might help us to determine the bottlenecks in the metabolism and increase the productivity.

We have demonstrated that synthetic autotrophic *K. phaffii* is able to produce organic acids by CO_2_ assimilation. In a CO_2_-based process, organic acids are suitable chemicals (46) to produce, as they have intermediate oxidation states which make them feasible products by using an oxidized substrate, like CO_2_. Our results support the metabolic engineering concept that pathways containing an irreversible step (like decarboxylation of cis-aconitate to itaconic acid) can run more efficiently compared to those where products can potentially serve as a carbon source (like lactic acid), especially when assimilation of the carbon source is limited and/or energy intensive (47). Therefore, target products have to be carefully evaluated for potential industrial application of such a system.

The titers we reached in shake flask cultivations (up to 2 g L^−1^) are still far from industrially feasible processes, but through further metabolic and process engineering steps, the synthetic CO_2_ assimilating *K. phaffii* can be a platform for production of value-added chemicals and form a chassis for a CO_2_-neutral or even CO_2_-negative bioprocesses.

## Materials and Methods

### Construction of the Strains.

The synthetic autotrophic *K. phaffii* strain described by Gassler et al. (13) was used as the host to build the organic acid producing strains. Cis-aconitate decarboxylase (*cadA*) and the mitochondrial carrier protein *mttA* from *Aspergillus terreus* (24) were expressed under methanol inducible promoters. For the lactic acid producing strains the *ldhL* gene of *Lactobacillus plantarum* was expressed under the control of the methanol inducible *AOX1* promoter. The deletion of *CYB2* was done using CRISPR-Cas9 and a homologous donor DNA repair template deleting the gene. All the plasmid constructions and transformations were performed by Golden Gate Assembly (GGA) and the CRISPR-Cas9 system described in Prielhofer et al. (25) and Gassler et al. (48). Integration to the correct loci and the correct gene deletion were verified by PCR. A list of the strains used in this study is given in Table 1.

**Table 1.** *K. phaffii* strains used in this study

Short Name	Genotype	References
Control	CBS7435 *aox1*Δ::p*AOX1_TDH3 +* p*FDH1_*PRK + p*ALD4_PGK1 das1*Δ::p*DAS1_*RuBisCO *+* p*PDC1_groEL* + p*RPP1B_groES* *das2*Δ::p*DAS2_TLK1 +* p*RPS2_TPI1*	Gassler et al. (13)
*cadA*	Control + p*AOX1_cadA*	This study
*cadA* + *mttA_strong_*	Control + p*AOX1_cadA* + p*FDH1_mttA*	This study
*cadA* + *mttA_medium_*	Control + p*AOX1_cadA* + p*POR1_mttA*	This study
*cadA* + *mttA_weak_*	Control + p*AOX1_cadA* + p*PDC1_mttA*	This study
*ldhL*	Control + p*AOX1_ldhL*	This study
*ldhL cyb2*Δ	Control + p*AOX1_ldhL cyb2*Δ	This study
Δ RuBisCO ita	Control ΔRuBisCO + p*AOX1_cadA* + p*POR1_mttA*	This study
Δ RuBisCO lac	Control ΔRuBisCO + p*AOX1_ldhL cyb2*Δ	This study
Control (eng strain)	Control (PRK 5 C > G)	Gassler et al. (22)
*ldhL* (eng strain)	Control (eng strain) + p*AOX1_ldhL*	This study
*ldhL cyb2*Δ (eng strain)	Control (eng strain) + p*AOX1_ldhL cyb2*Δ	This study
*cadA* (eng strain)	Control (eng strain) + p*AOX1_cadA*	This study

### Shake Flask Cultivations.

A two-phase screening protocol was used in this study. First, a preculture in YPG (yeast extract 10 g L^−1^, soy peptone 20 g L^−1^, glycerol 20 g L^−1^) was conducted for 22 to 24 h until enough biomass was reached. Then, the preculture volume required for the inoculation was washed twice and cells were transferred into the main culture with the target starting OD_600_ (4, 8, 10, 12, 16, or 20) for the cultivation under autotrophic conditions where cells were cultivated in 100 mL narrow-neck flasks at 30 °C with 5% or 10% constant CO_2_ supply in buffered YNB (3.4 g L^−1^, pH 6) supplemented with 10 g L^−1^ (NH_4_)_2_SO_4_ as the nitrogen source. Cells were induced with 0.5% (vol/vol) methanol at the start of the main culture, and further adjusted to 1% (vol/vol) methanol from there on until the end of the cultivations. Cell growth (OD_600_) was monitored throughout the cultivation and extracellular metabolite concentrations (methanol, lactic acid, itaconic acid, and glycolic acid) were measured by high-performance liquid chromatography (HPLC) and culture volume was corrected for evaporation by the addition of water.

Cultivations performed with lactic acid as sole carbon source were performed by using 100 mL narrow-neck flasks at 25 °C in buffered YNB (3.4 g L^−1^, pH 6) supplemented with 10 g L^−1^ (NH_4_)_2_SO_4_ as the nitrogen source and 10 g L^−1^ lactic acid as carbon source. Cells were inoculated with the target starting OD_600_ of 4 and samples for optical density and HPLC (for remaining lactic acid concentration) were taken regularly.

### HPLC Measurements.

A Biorad Aminex HPX-87H HPLC column (300 × 7.8 mm) was used for the HPLC measurements. H_2_SO_4_ at a concentration of 4 mmol L^−1^ was used as mobile phase, with a 0.6 mL min^−1^ flow rate at 60 °C. Itaconic acid was measured with a photodiode array detector (SPD-M20A, Shimadzu) at 254 nm. Lactic acid, glycolic acid, glycerol, and methanol concentrations were measured with a refraction index detector (RID-10A, Shimadzu). After centrifugation, the supernatant of each sample was mixed with H_2_SO_4_ (*c* = 40 mmol L^−1^) resulting in a final concentration of 4 mM. Samples were vortexed and centrifuged at full speed (16,100 g) for 5 min at room temperature. After centrifugation, they were filtered using a 0.22 µm filter into the vials for the HPLC analysis.

### Bioreactor Cultivations.

Bioreactor cultivations were performed using 1.4 L DASGIP reactors (Eppendorf). Cultivations were conducted using YNB media supplemented with 10 g L^−1^ (NH_4_)_2_SO_4_ as the nitrogen source and buffered using 100 mmol L^−1^ phosphate buffer at pH 6 at 30 °C. pH was controlled using 2 mol L^−1^ NaOH. Dissolved oxygen concentration was controlled by adjusting the stirrer speed and inlet gas flow whereby 200 rpm and 6 sL h^−1^ were the minimal setpoints.

The itaconic acid fermentations were performed as follows: bioreactors were inoculated from a YPG preculture to an OD of 1 and a glycerol batch in YNB media was performed to reach an end biomass of approximately OD 20 and 30, respectively, based on a yield on glycerol of 0.5. After the batch-end cultures were fed with 0.5% (vol/vol) methanol and supplied with 10% CO_2_ in the inlet gas. After the first sample (∼16 h) methanol concentration was adjusted to 1% (vol/vol). From this time on samples were taken daily including OD, DCW, and HPLC samples. After each sampling the methanol concentration was adjusted to 1% (vol/vol). The lactic acid fermentation was performed as follows: bioreactors were inoculated from a YPG preculture to an OD of 2. The autotrophic cultivation was performed from beginning on using 5% CO_2_ in the inlet gas and 0.5% methanol. After the first sample methanol concentration was increased to 1%. Samples were taking daily including OD and HPLC measurements. The methanol concentration was adjusted to 1% after 2 or 3 d.

Overall specific growth rate and production rates of the producer strains in the shake flasks and bioreactor cultivations were calculated according to Eqs. **1** and **2**:

[1] µ=lnX2X1t2−t1

[2] $$q_{P}=\;\text{µ}*Y_{P/X}$$

µ is the specific growth rate (h^−1^), X is the cell concentration (g L^−1^) at t (h) of the cultivation, q_P_ is the specific production rate (mg g^−1^ h^−1^), Y_P/X_ is the product yield (mg g^−1^ h^−1^).

For the nongrowing strains, production rates were calculated according to Eq. **3**:

[3] $$q_{P}=\frac{P_{2}-P_{1}}{\frac{X_{2}-X_{1}}{2}*(t_{2}-t_{1})}$$

q_P_ is the specific production rate (mg g^−1^ h^−1^), P is the product concentration (mg L^−1^) at t (h) of the cultivation, X is the cell concentration (g L^−1^) at t (h) of the cultivation.

### ^13^C Labeling.

The incorporation of carbon from CO_2_ into the produced organic acids was determined with a reverse ^13^C labeling approach. To fully label biomass with ^13^C, cells were grown on YNB with ^13^C glycerol until the entire glycerol was depleted (>10 generations). After washing, cells were transferred into YNB and induced with ^13^C labeled methanol as described above. The cells were fed with 5% ^nat^CO_2_ (CO_2_ with a natural isotope distribution) for growth and production. Samples were taken at Day 3, Day 5, and Day 8. After harvesting, cells were centrifuged at 4,000 g for 5 min at 4 °C and supernatants were kept at −20 °C until further analysis with GC-TOFMS (gas chromatography with a coupled time of flight mass spectrometer). Itaconic and lactic acid concentrations (*SI Appendix*, Fig. S4) were determined by HPLC as described above. Three biological replicates were used for isotopologue distribution analysis.

### Elemental Analysis–Isotope-Ratio Mass Spectrometry for ^13^C Biomass Content.

To verify ^13^C labeling of the biomass a fraction of the cells was harvested after ^13^C glycerol cultivation and centrifuged at 4 °C for 5 min at 4,000 g. Biomass corresponding to ∼0.5 mg DCW was washed once with filter sterilized 0.1 mol L^−1^ HCl and twice with sterile distilled water. The ^13^C/^12^C ratio was measured with an isotope-ratio mass spectrometer coupled to an elemental analyzer. Elemental analysis–isotope-ratio mass spectrometry (EA–IRMS) measurements were carried out by Imprint Analytics GmbH, Austria as described in Gassler et al. (13). Samples were kept at −20 °C until analysis.

### GC-TOFMS Isotopologue Distribution Analysis of Lactic Acid and Itaconic Acid in the Culture Supernatant.

As the workflow published by Mairinger et al. (49), which is used routinely in our laboratory for the determination of ^13^C labeling patterns, includes neither lactic nor itaconic acid as target analytes, a GC-EI-TOFMS method was developed for the selective determination of the ^13^C labeling patterns of the two analytes of interest in culture supernatants. More specifically, derivatization and separation strategies had to be established. In brief, 0.5 mL of the supernatants were filtered using 10 kDa spin filters (Millipore Amicon Ultra). Aliquots of 9 to 120 µL were dried in 1.5 mL chromatography crimp vials supplied with 400 µL flat bottom inserts, resulting in on-column amounts of 10 to 12 pmol for itaconic acid and 20 to 22 pmol for lactic acid (estimation of aliquot volume needed was based on HPLC data (*SI Appendix*, Fig. S4). Immediately after drying the samples were stored on a cooled rack (7 °C) of the Gerstel MPS2 sample preparation robot until just in time derivatization and injection into the GC-(Q)TOFMS. For automated tert-butyldimethylsilyl derivatization, 40 µL of water free pyridine (Sigma Aldrich) and 40 µL of *N*-tert-butyldimethylsilyl-*N*-methyltrifluoroacetamide with 1% tert-butyldimethylchlorosilane (Sigma Aldrich) were added and the samples were incubated at 85 °C for 60 min. 1 µL of derivatized sample was injected into an Ultra Inert straight split liner of an Agilent split/splitless injector in split mode (250 °C, septum purge flow 3 mL min^−1^). The split ratio was set to 1:5, 1:10 or 1:50 targeting the desired on-column amount, hence depending on analyte concentration and aliquot volume. The gas chromatographic system (Agilent 7890B GC, Agilent Technologies Inc.) was equipped with 1) a nonpolar deactivated precolumn [3 m × 0.25 µm inner diameter (i.d.), Phenomenex], followed by 2) the analytical column [HP-5MS UI (5% phenylpolysiloxane, 30 m × 0.25 mm × 0.25 µm, Agilent] and 3) a nonpolar deactivated restrictor column (3 m × 0.18 µm i.d., Phenomenex). All columns were connected with purged ultimate unions, (Agilent Technologies Inc.). The carrier gas was helium (purity >99.999%) at a constant flow of 1.2 mL min^−1^ for the precolumn, 1.3 mL min^−1^ for the analytical column and 1.5 mL min^−1^ for the post column. For the separation the following GC-temperature program was employed (total run time 29.3 min): 95 °C for 1 min, 10 °C min^−1^ to 135 °C, 3 °C min^−1^ to 170 °C, 30 °C min^−1^ to 310 °C, hold for 8 min. The transfer line temperature was set to 280 °C.

As mass analyzer, an Agilent Technologies 7200B Q-TOF mass spectrometer (Agilent Technologies Inc.) was used in TOF mode with electron ionization (70 eV, 230 °C). The solvent delay was set to 5.5 min and the ion source was turned off during elution of the highly concentrated phosphate derivative (17 to 19 min).

Retention times of itaconic and lactic acid, mass/charge ratios evaluated for ^13^C isotopologue distribution analysis and the respective extraction windows of the fragments are listed in *SI Appendix*, Table S3.

For peak integration, the software MassHunter Workstation, Quantitative Analysis (Version 10.1, Agilent Technologies Inc.) was used in centroid and profile mode, using a symmetric extraction window of ± 50 ppm. The data interpreted in this paper are those obtained by evaluation of the fragment [M-CH_3_] ^+^ in centroid mode (for further information on method optimization see *SI Appendix*). To receive the isotopologue distribution of the carbon backbone only, the peak areas were corrected for naturally distributed heavy stable isotopes using the ICT correction toolbox (50). This program corrects for Si, S, O, N and H isotopes of the derivatized molecule as well as the C isotopes stemming from derivatization (i.e., all C atoms apart from backbone C atoms).

## Supplementary Material

Supplementary File

## Data Availability

All study data are included in the article and/or supporting information.
