# Supplementary material for: Conversion of CO2 into organic acids by engineered autotrophic yeast
Source: Proc Natl Acad Sci U S A. 2022 Nov 16;119(47):e2211827119. doi: 10.1073/pnas.2211827119 (PMC9704707; doi:10.1073/pnas.2211827119)
Supplement: Supplementary File [file pnas.2211827119.sapp.pdf]

# Supplementary Information

## Production of organic acids from $CO_2$ by engineered autotrophic yeast *Komagataella phaffii*

Michael Baumschabl[1,2,§], Özge Ata[1,2,§], Bernd M. Mitic[2,3], Lisa Lutz[1,2], Thomas Gassler[1,2,+], Christina Troyer[3], Stephan Hann[1,3], Diethard Mattanovich[1,2,*]

[1] Austrian Centre of Industrial Biotechnology (ACIB), Vienna, Austria

[2] Institute of Microbiology and Microbial Biotechnology, Department of Biotechnology, University of Natural Resources and Life Sciences (BOKU), Vienna, Austria

[3] Department of Chemistry, Institute of Analytical Chemistry, University of Natural Resources and Life Sciences, (BOKU), Vienna, Austria

[+] Present address: Institute of Microbiology, ETH Zurich, Zurich, Switzerland

[§] These authors contributed equally to this work

* Corresponding author

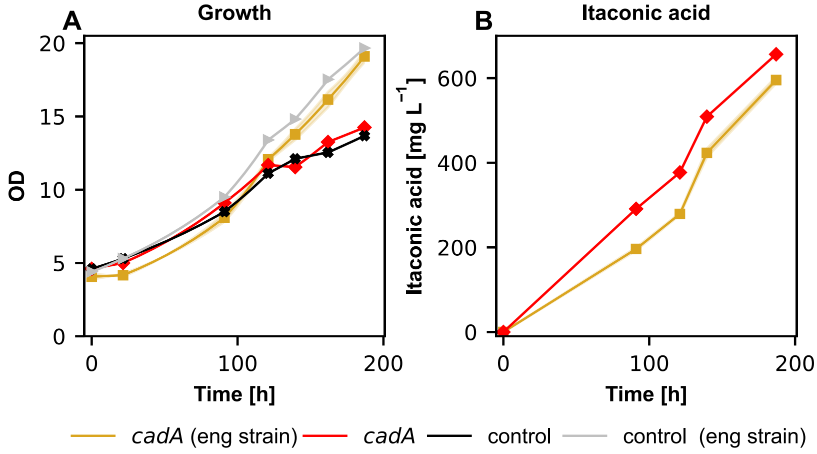


**Supplementary Figure S1. Reverse engineered *K. phaffii*** (1) **performed worse than the parent producing strain.** (**A**) Growth profile, (**B**) itaconic acid production profile. Time axis corresponds to the production phase under autotrophic conditions. At least three biological replicates were used in the screening to monitor the producing strains. Shades represent the standard deviations (±). eng strain: Reverse engineered autotrophic *K. phaffii* strain.

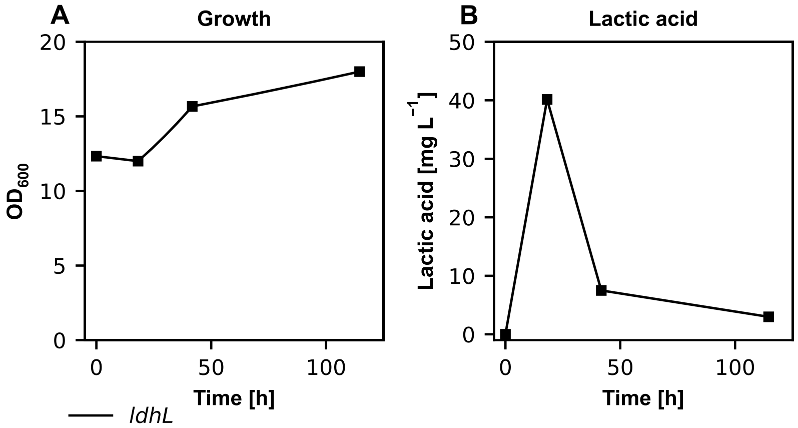


**Supplementary Figure S2. High biomass concentrations led to re-assimilation of the produced lactic acid.** (**A**) Growth profile and (**B**) lactic acid production profile of the autotrophic *K. phaffii* strain with a L-lactate dehydrogenase integrated under the control of the methanol inducible *AOX1* promoter. Time axis corresponds to the production phase under autotrophic conditions. Biomass was grown in a glycerol batch followed by the production phase under autotrophic conditions.

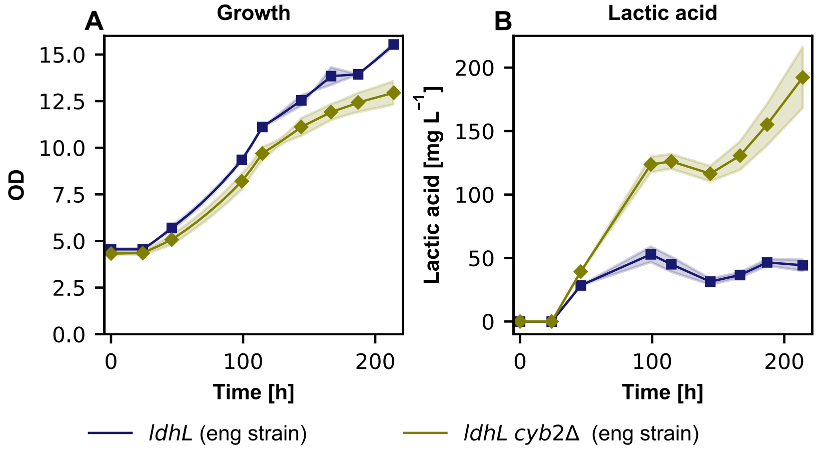


**Supplementary Figure S3. Lactic acid production was not improved by using the reverse engineered *K. phaffii* strain** (1)**.** (**A**) Growth profile, (**B**) lactic acid production profile. Time axis corresponds to the production phase under autotrophic conditions. At least three biological replicates were used in the screening to monitor the producing strains. Shades represent the standard deviations (±).eng strain: reverse engineered autotrophic *K. phaffii* strain.

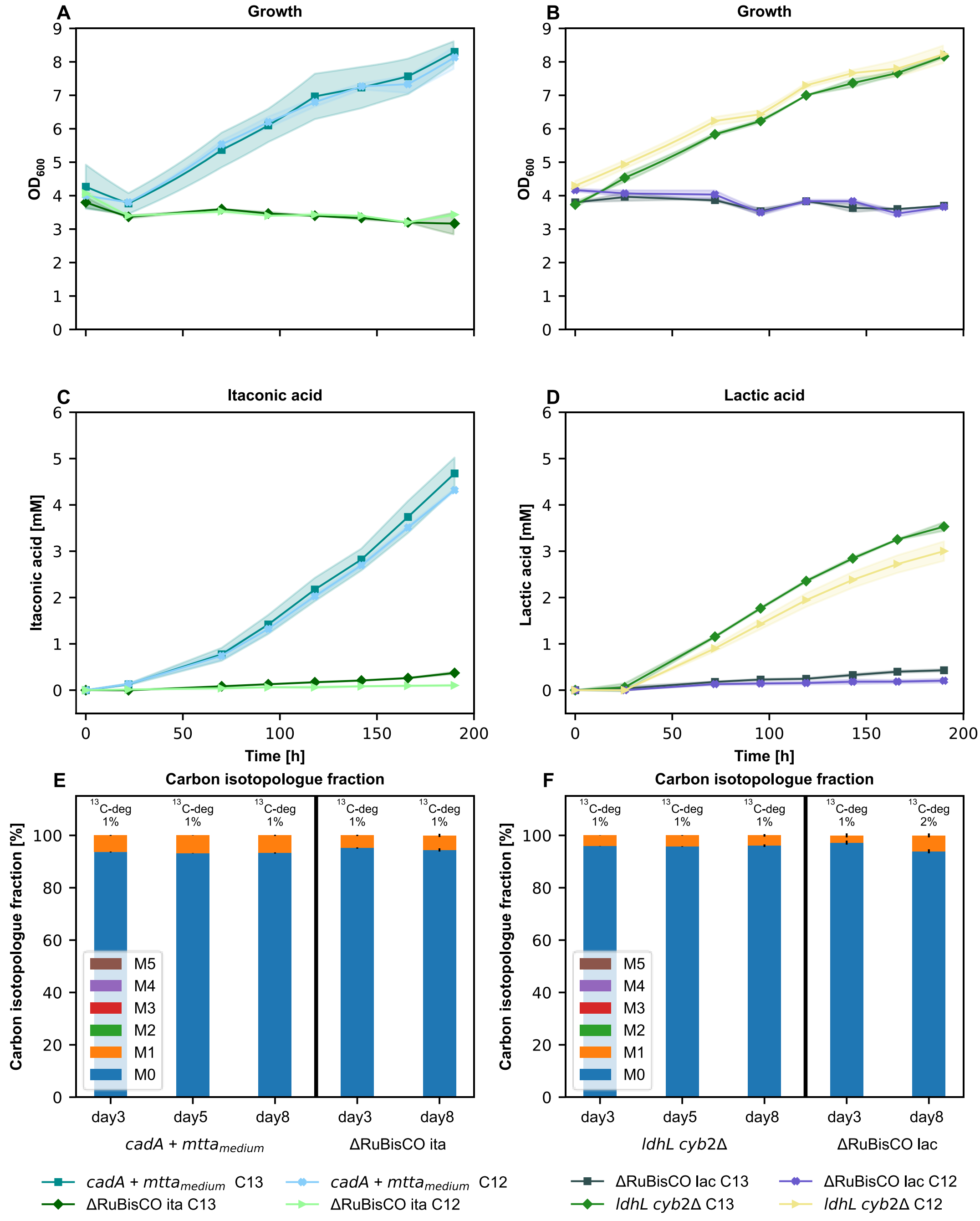


**Supplementary Figure S4.** Control cultivations performed with unlabeled glycerol and methanol in the $^{13}C$ labeling experiment showed similar profiles to the labelled cultures. **(A)** Growth profile of unlabeled and labeled itaconic acid production and non-grower strain, **(B)** growth profile of unlabeled and labeled lactic acid production and non-grower strain, **(C)** itaconic acid production profile of unlabeled and labeled producer and non-grower strain, **(D)** lactic acid production profile of unlabeled and labeled producer and non-grower strain, **(E)** carbon isotopologue distribution in the produced itaconic acid, **(F)** carbon isotopologue distribution in the produced lactic acid. M# denotes the number of $^{13}C$ carbons in the respective organic acid and $^{13}C$-deg indicates the $^{13}C$ labeling degree of every sample. Time axis corresponds to the production phase under autotrophic conditions. At least three biological replicates were used in the screening to monitor the strains. Time axis corresponds to the production phase under autotrophic conditions. Shades represent the standard deviations (±).

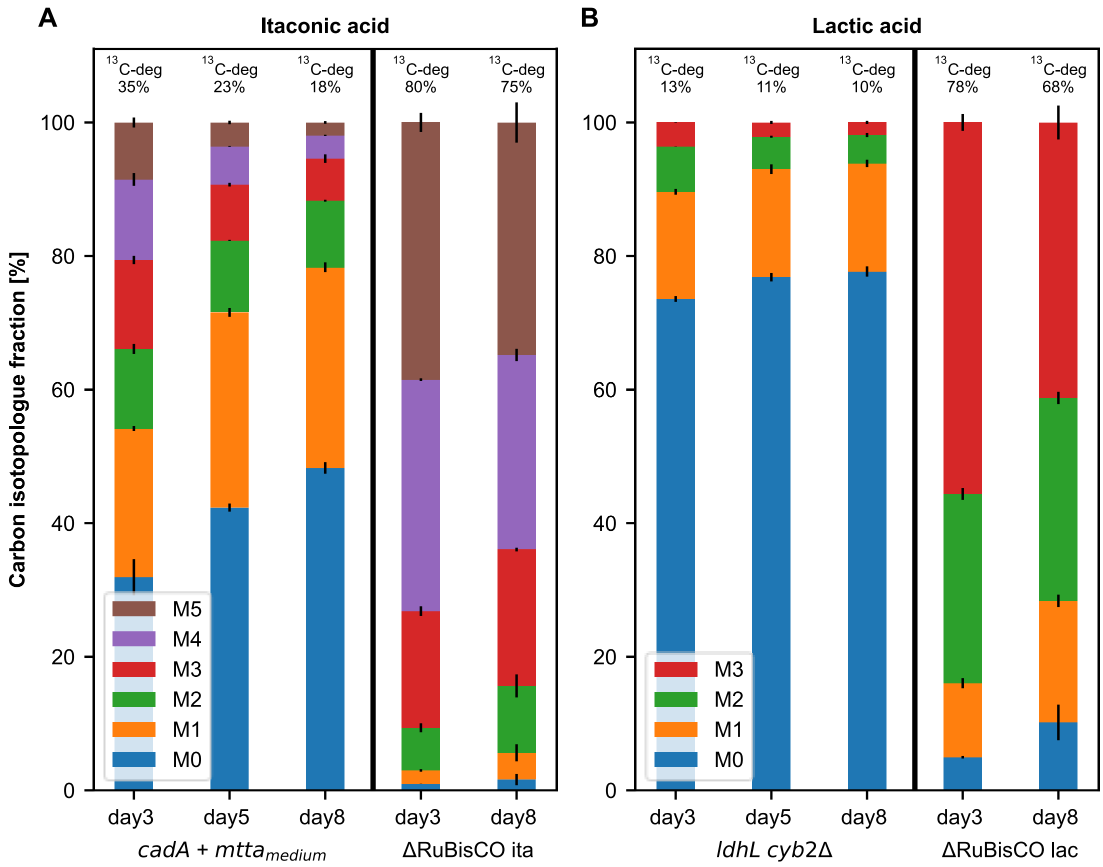


**Supplementary Figure S5.** Isotopologue distribution of the carbon atoms of the reversed labeled samples in the produced (**A**) itaconic acid and (**B**) lactic acid. M# denotes the number of $^{13}$C carbons in the respective organic acid and $^{13}$C-deg indicates the $^{13}$C labeling degree of every sample. Error bars indicate the standard deviation of three biological replicates.

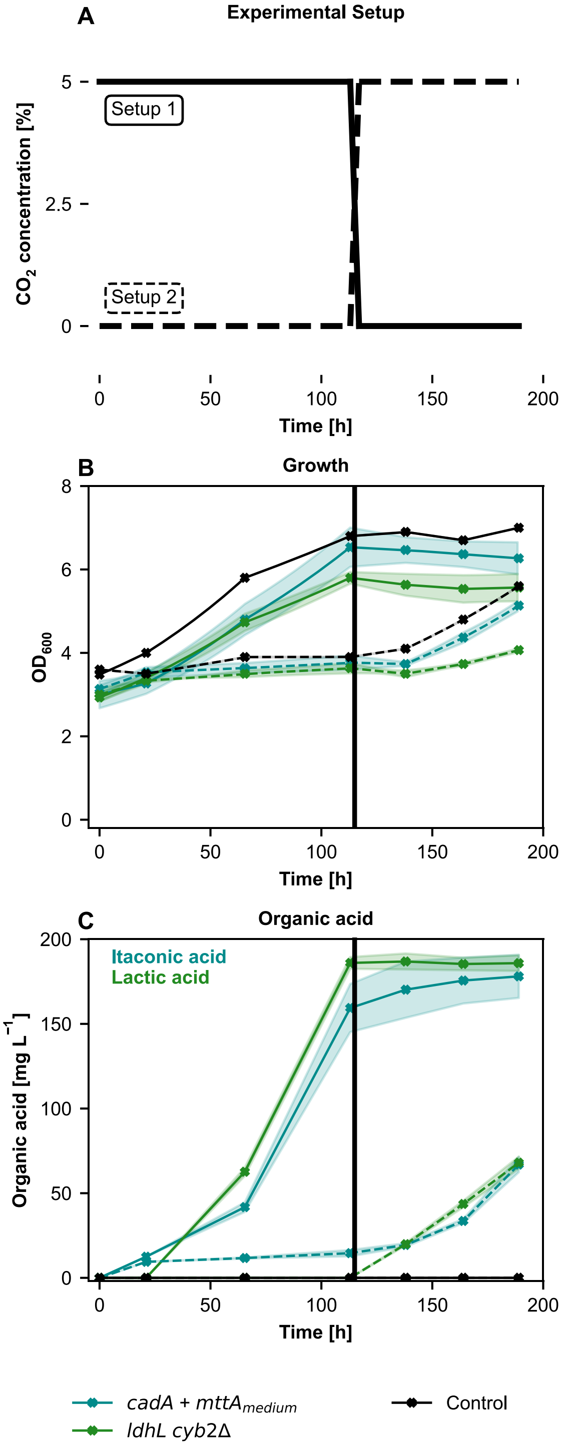


**Supplementary Figure S6.** Production of organic acids in the synthetic autotrophic *K. phaffii* strain depends on elevated $CO_2$ concentration. (**A**) Setup 1: cultures are incubated after the start of the production phase at 5% $CO_2$ and after the sampling point at 115h put to ambient $CO_2$ concentration. Setup 2: cultures are incubated after the start of the production phase at ambient $CO_2$ concentration and after the sampling point at 115h put to 5% $CO_2$. (**B**) Growth profiles of cultures cultivated according to setup 1 are shown with a solid line and cultures cultivated according to setup 2 are shown with a dashed line. (**C**) Organic acid production profiles of cultures cultivated using setup 1 (solid line) and cultures cultivated using setup 2 (dashed line). Time axis corresponds to the production phase under autotrophic conditions. Shades represent the standard deviation of 3 biological replicates (±).

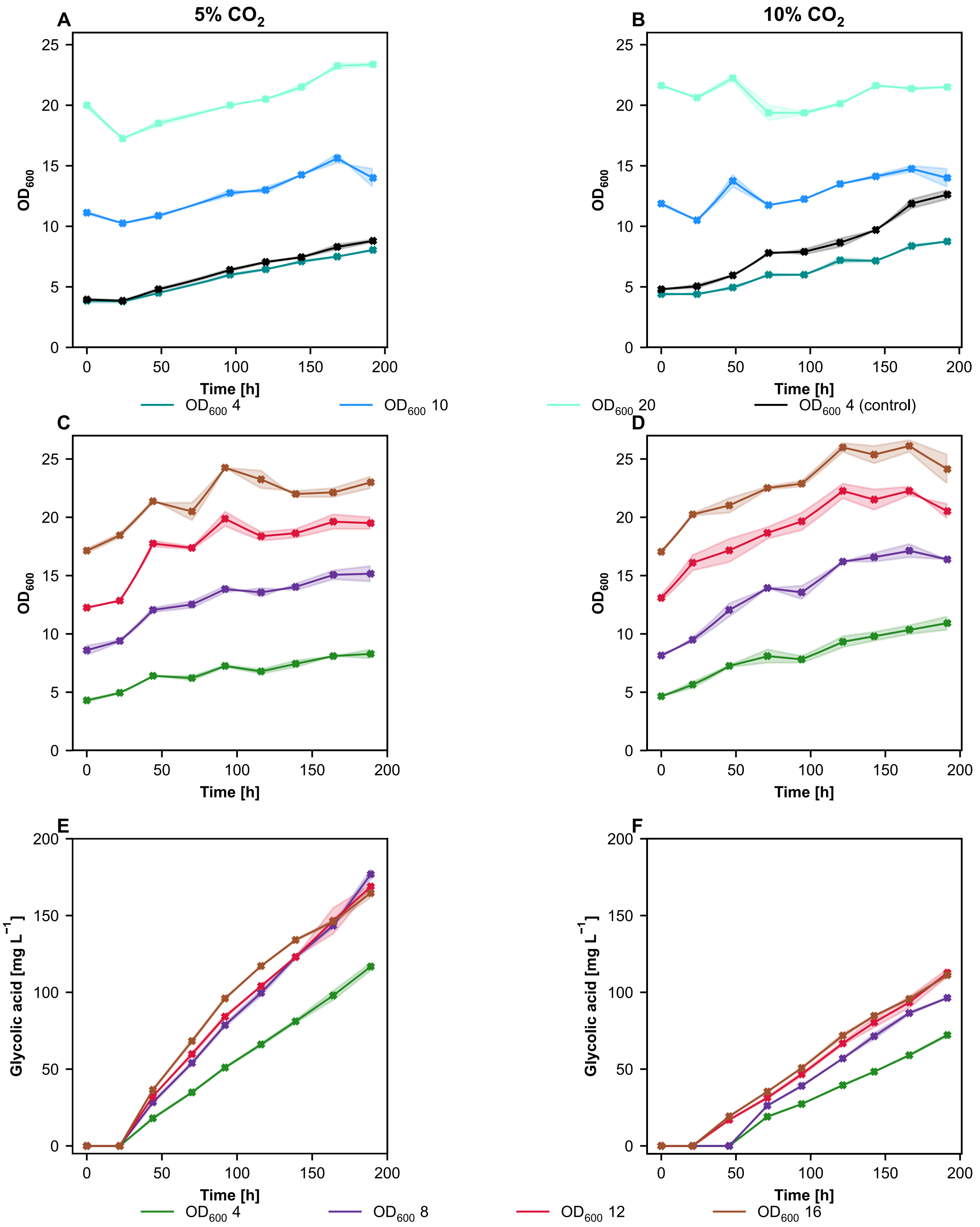


**Supplementary Figure S7.** Modification of process conditions shows different results for the different products. (**A**) Growth profiles of the itaconic acid production strain and the control using different starting biomass at 5% $CO_2$ concentration. (**B**) Growth profiles of the itaconic acid production strain and the control using different starting biomass at 10% $CO_2$ concentration. (**C**) Growth profiles of the lactic acid production strain using different starting biomass at 5% $CO_2$ concentration. (**D**) Growth profiles of the lactic acid production strain using different starting biomass at 10% $CO_2$ concentration. (**E**) Glycolic acid production profiles of the different starting biomasses of the lactic acid production strain at 5% $CO_2$ concentration. (**F**) Glycolic acid production profiles of the different starting biomasses of the lactic acid production strain at 10% $CO_2$ concentration. Time axis corresponds to the production phase under autotrophic conditions. Shades represent the standard deviations of biological replicates (±).

**Supplementary Table S1.** Specific growth rates, specific production rates, and yields of the strains used in this study. The values given in ranges belong to multiple screenings.

| | **Strain** | **$CO_2$ Supply (%)** | **Starting $OD_{600}$ in the production phase** | **μ ($h^{-1}$)** | **$q_P$ (mg $g^{-1}$ DCW $h^{-1}$)** | **Final Titers (mg $L^{-1}$)** |
|---|---|---|---|---|---|---|
| **Itaconic Acid** | *cadA* | 5 | 3-4 | 0-006-0.007 | 1.98-2.26 | 517-530 |
| | *cadA* + $mttA_{weak}$ | 5 | 3-4 | 0.005 | 2.09 | 562 |
| | *cadA* + $mttA_{medium}$ | 5 | 3-4 | 0.004-0.005 | 2.65-3.19 | 553-754 |
| | *cadA* + $mttA_{strong}$ | 5 | 3-4 | 0.001 | 1.32 | 195 |
| | *cadA* + $mttA_{medium}$ | 5 | 10 | 0.003 | 1.90 | 908 |
| | *cadA* + $mttA_{medium}$ | 5 | 20 | 0.002 | 1.34 | 1025 |
| | *cadA* + $mttA_{medium}$ | 10 | 4 | 0.004 | 3.90 | 886 |
| | *cadA* + $mttA_{medium}$ | 10 | 10 | 0.002 | 3.66 | 1611 |
| | *cadA* + $mttA_{medium}$ | 10 | 20 | No growth | 2.73 | 1980 |
| | *cadA* + $mttA_{medium}$ | 10 | 20 (DO = 8%) | No growth | 0.74 | 525 |
| | *cadA* + $mttA_{medium}$ | 10 | 30 (DO = 20%) | No growth | 0.16 | 242 |
| | Δ RuBisCO ita | 5 | 4 | No growth | 0.10-0.39 | 18-48 |
| | *cadA* (eng strain) | 5 | 4 | 0.008-0.009 | 1.39-1.49 | 491-521 |
| **Lactic Acid** | *ldhL* | 5 | 3-4 | 0.005 | 0.85 | 196 |
| | *ldhL cyb2*Δ | 5 | 3-4 | 0.003-0.005 | 1.20-2.79 | 250-640 |
| | *ldhL cyb2*Δ | 5 | 8 | 0.003 | 0.72 | 316 |
| | *ldhL cyb2*Δ | 5 | 12 | 0.002 | 0.31 | 183 |
| | *ldhL cyb2*Δ | 5 | 16 | 0.002 | 0.23 | 172 |
| | *ldhL cyb2*Δ | 10 | 4 | 0.005 | 1.49 | 413 |
| | *ldhL cyb2*Δ | 10 | 8 | 0.004 | 0.74 | 331 |
| | *ldhL cyb2*Δ | 10 | 12 | 0.002 | 0.42 | 260 |
| | *ldhL cyb2*Δ | 10 | 16 | 0.002 | 0.31 | 239 |
| | *ldhL cyb2*Δ (Ferm 21% oxygen) | 5 | 2 | 0.002 | 0.76 | 266 |
| | *ldhL cyb2*Δ (Ferm 5% oxygen) | 5 | 2 | 0.004 | 0.22 | 140 |
| | Δ RuBisCO lac | 5 | 4 | No growth | 0.11-0.25 | 15-35 |
| | *ldhL* (eng strain) | 5 | 4 | 0.007 | 0.40 | 44 |
| | *ldhL cyb2*Δ (eng strain) | 5 | 4 | 0.006 | 0.62 | 192 |

**Supplementary Table S2.** Stoichiometric model for the calculation of expected $^{13}C$ labeling degree using isotopomer network compartmental analysis (INCA) (2).

| Reaction ID | Reaction equation with carbon transition |
|---|---|
| **R1** | CO2up.int (a) -> CO2up (a) |
| **R2** | CO2up (a) + CO2up (b) + CO2up (c) -> G3P (abc) |
| **R3** | G3P (abc) -> 2PG (abc) |
| **R4** | 2PG (abc) -> Pyr (abc) |
| **R5** | Pyr (abc) -> AcCoA (bc) + CO2pro (a) |
| **R6** | Pyr (abc) + CO2up (d) -> OAA (abcd) |
| **R7** | OAA (abcd) + AcCoA (ef) -> Cit (dcbfea) |
| **R8** | Cit (abcdef) -> Cisa (abcdef) |
| **R9*** | Cisa (abcdef) -> CO2pro (a) + Ita (bcdef) |
| **R10** | Cisa (abcdef) -> ICit (abcdef) |
| **R11** | ICit (abcdef) -> AKG (abcde) + CO2pro (f) |
| **R12** | AKG (abcde) -> SucCoA (bcde) + CO2pro (a) |
| **R13** | SucCoA (abcd) -> Suc (abcd) |
| **R14** | Suc (abcd) -> Fum (abcd) |
| **R15** | Fum (abcd) -> Mal (abcd) |
| **R16** | Mal (abcd) -> OAA (abcd) |
| **R17** | CO2pro (a) -> CO2pro.ext |
| **R18*** | Ita (abcde) -> Ita.ext (abcde) |
| **R19** | 0.06332*AcCoA + 0.03415*AKG + 0.0002588*G3P + 0.02089*OAA + 0.06562*Pyr -> Biomass + 0.02641*CO2pro |
| **R20**** | Pyr (abc) -> Lac (abc) |
| **R21**** | Lac (abc) -> Lac.ext (abc) |

* Reactions included only for itaconic acid producer strains

** Reactions included only for lactic acid producer strains

**Supplementary Table S3.** Retention times of lactic and itaconic acid, evaluated electron ionization fragments and isotopologues, m/z values of isotopologues extracted for $^{13}C$ isotopologue distribution analysis.

| Name of analyte fragment* | RT (min) | Fragment evaluated** | Isotopologue*** | m/z |
|---|---|---|---|---|
| Lac-M-57 2TBDMS | 8.54 | $[M-C_4H_9]^+$ | M0 | 261.1337 |
| | | | M1 | 262.1370 |
| | | | M2 | 263.1404 |
| | | | M3 | 264.1437 |
| Lac-M-15 2TBDMS | 8.54 | $[M-CH_3]^+$ | M0 | 303.1806 |
| | | | M1 | 304.1840 |
| | | | M2 | 305.1873 |
| | | | M3 | 306.1907 |
| Ita-M-57 2TBDMS | 15.89 | $[M-C_4H_9]^+$ | M0 | 301.1286 |
| | | | M1 | 302.1319 |
| | | | M2 | 303.1354 |
| | | | M3 | 304.1387 |
| | | | M4 | 305.1420 |
| | | | M5 | 306.1454 |
| Ita-M-15 2TBDMS | 15.89 | $[M-CH_3]^+$ | M0 | 343.1755 |
| | | | M1 | 344.1789 |
| | | | M2 | 345.1822 |
| | | | M3 | 346.1856 |
| | | | M4 | 347.1890 |
| | | | M5 | 348.1923 |
| | | | | |

* the names of the derivatized metabolites specify the number of tertbutyldimetylsilyl (TBDMS) groups

** the number of the isotopologue denotes the number of $^{13}C$ atoms in the backbone

*** electron ionization fragments used for data evaluation: $[M-CH_3]^+$: abstraction of $CH_3$, $[M-C_3H_9]^+$: abstraction of $C_3H_9$

## Supplementary Methods

***Optimization of GC-EI-TOFMS data evaluation for isotopologue distribution analysis***

For each analyte two fragments, namely M-57 (= $[M\text{-}C_4H_9]^+$) and M-15 (= $[M\text{-}CH_3]^+$), were evaluated in both profile and centroid mode using a symmetric extraction window of ± 50 ppm, resulting in a total of four different evaluation methods.

For the assessment of the different evaluation methods, the $^{13}C$ labeling degree was calculated based on the isotopologue fractions as defined by Mairinger et al. (3) (Equation S1 & Equation S2):

*Equation S1: $^{13}C$ labeling degree*

$$^{13}C\ labeling\ degree\ [\%] = \frac{\sum_{i=0}^{n} IF_i * i}{n * \sum_{i=0}^{n} IF_i}$$

*Equation S2: isotopologue fraction of isotopologue i*

$$IF_i\ [\%] = \frac{A_i}{\sum_{i=0}^{n} A_i}$$

n = number of carbon atoms in metabolite backbone
$IF_i$ = isotopologue fraction of isotopologue i
$A_i$= peak area of isotopologue i

The calculated $^{13}C$ labeling degrees are displayed in Supplementary Figures S7 to S8 for itaconic acid and Supplementary Figures S9 to S10 for lactic acid. Data are shown for all samples evaluated for this study, $^{nat}C$ samples as well as $^{13}C$ samples.

For samples cultivated on $^{nat}C$ media (media with a natural carbon isotope distribution), data for a total of 15 biological replicates were available, more specifically 3 replicates each for 2 time points for the knock out strain and 3 time points for the producing strain with the RuBisCO gene, resulting in a total of 5 groups of n=3 replicates.

The measured $^{13}C$ labeling degree of these 15 $^{nat}C$ samples was compared to the expected $^{13}C$ labeling degree deduced from the natural carbon isotope distribution (see Supplementary Figure S8 for itaconic acid and Supplementary Figure S10 for lactic acid).

For itaconic acid the evaluation of the M-57 in profile and M-15 in centroid mode shows no significant difference to the expected value, nevertheless M-57 in profile mode had to be rejected due to the high standard deviation. Generally, all results show higher standard deviation for $^{nat}C$ samples, as higher mass isotopologues are of low intensity in these samples.

For the $^{13}C$ samples (see Supplementary Figure S9), whose $^{13}C$ labeling degree cannot be compared to any expected values as it is the case for the $^{nat}C$ samples, Analysis of Variance (ANOVA) was applied for each replicate group in order to check for statistically significant differences between the results obtained with the different evaluation methods ($\alpha$=0.05, p-values in Figure legend). For all 5 replicate groups of the $^{13}C$ samples no significant difference could be observed for the four different evaluation methods.

In summary, the evaluation of the M-15 itaconic acid fragment in centroid mode gave best results in terms of deviation from the expected value and precision under repeatability conditions of measurement (see Supplementary Figure S8). Thus all data presented for itaconic acid in this paper are based on that data evaluation method (see Experimental).

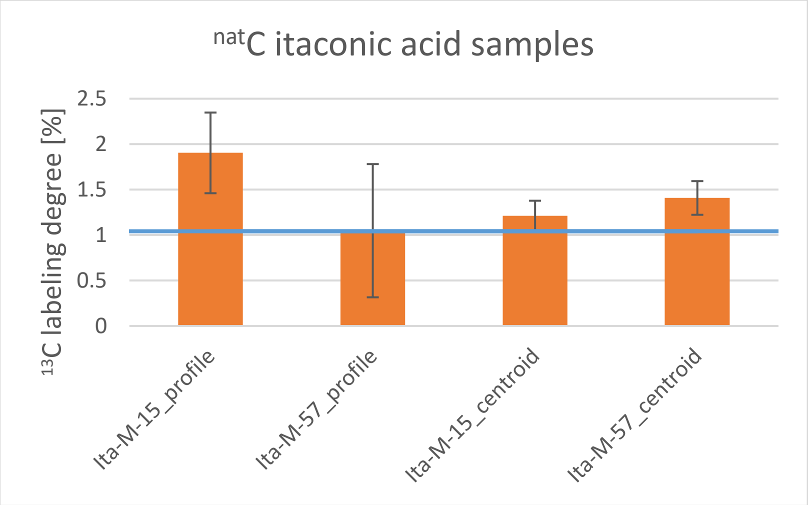


**Supplementary Figure S8.** $^{13}C$ labeling degree of itaconic acid (average ± standard deviation) obtained using four different data evaluation methods (orange bars) for n=15 $^{12}C$ samples (5 groups of 3 biological replicates each). The blue line depicts the expected value for the $^{nat}C$ samples.

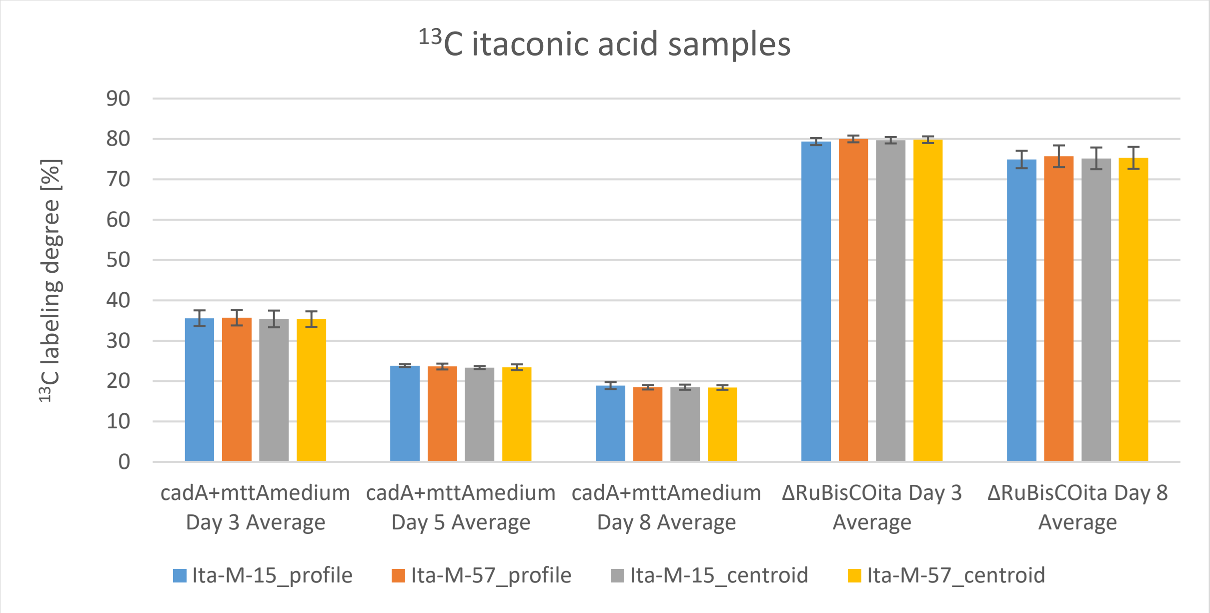


**Supplementary Figure S9.** $^{13}C$ labeling degree of itaconic acid (average ± standard deviation) in 5 groups (different time points/strains) of $^{13}C$ samples (n=3 biological replicates) evaluated with four different evaluation methods; p-values of ANOVA for each group: cadA+mttA$_{medium}$ Day 3: 0.996, cadA+mttA$_{medium}$ Day 5: 0.758, cadA+mttA$_{medium}$ Day 8: 0.811, ΔRuBisCO ita Day 3: 0.789, ΔRuBisCO ita Day 8: 0.985.

The same approach was used for the comparison of the evaluation methods for lactic acid.

For the $^{nat}C$ samples (n=15, see Supplementary Figure S10) both data evaluation methods based on fragment M-15 showed comparable deviation from the expected value and variance.

In contrast to itaconic acids evaluation of the $^{13}C$ samples (Supplementary Figure S11) shows no significant differences between the four sample evaluation methods (p-values for ANOVA in Figure legend) for lactic acid.

As both data evaluation methods based on the fragment M-15 can be regarded as not significantly different in terms of deviation from the expected value and precision under repeatability conditions of measurement, data evaluation for lactic acid was also carried out in centroid mode evaluating the fragment M-15.

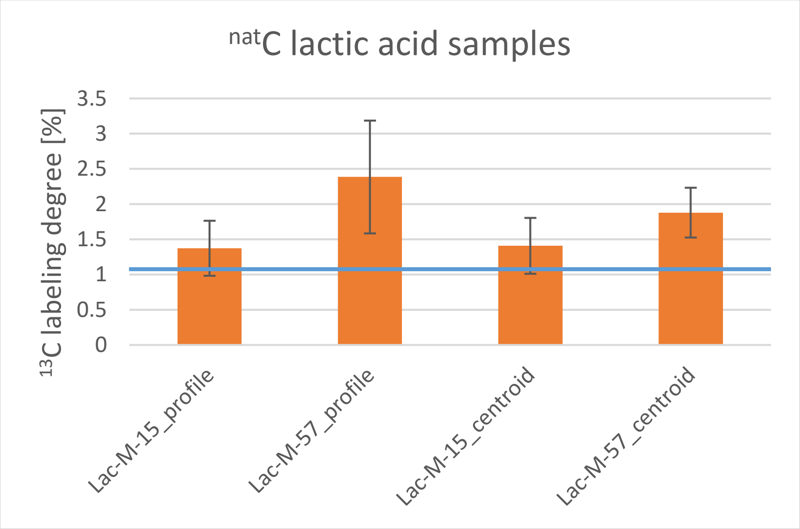


**Supplementary Figure S10.** $^{13}C$ labeling degree of lactic acid (average ± standard deviation) obtained using four different data evaluation methods (orange bars) for n=15 $^{12}C$ samples (5 groups of 3 biological replicates each). The blue line depicts the expected value for the $^{nat}C$ samples.

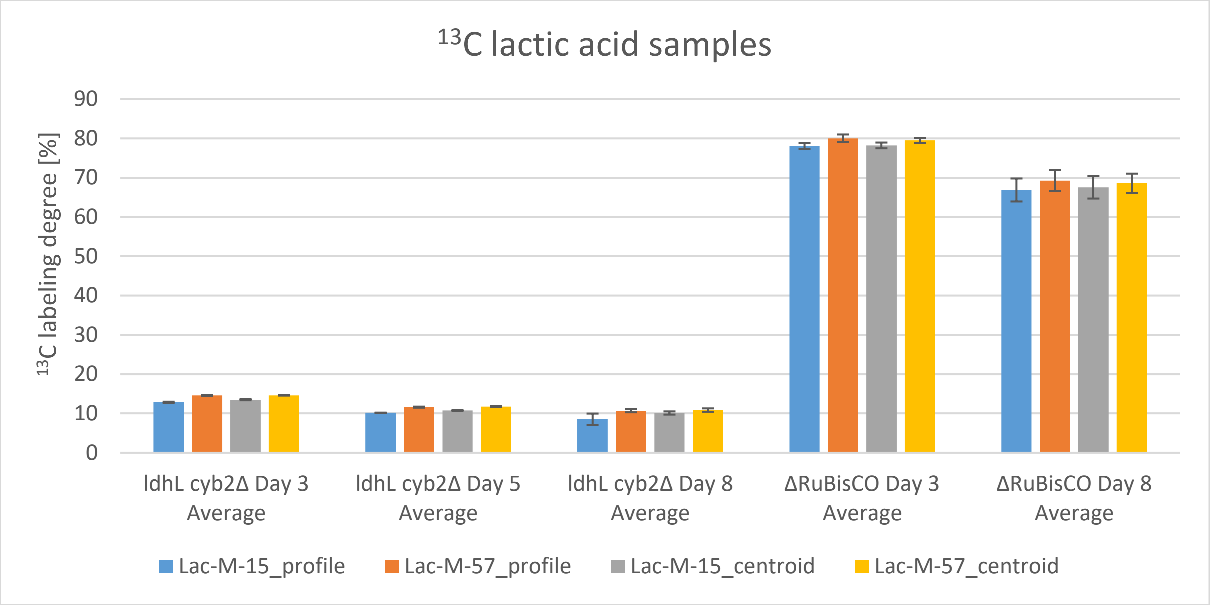


**Supplementary Figure S11.** $^{13}$C labeling degree of lactic acid (average ± standard deviation) in 5 groups (different time points/strains) of $^{13}$C samples (n=3 biological replicates) evaluated with four different evaluation methods; p-values of ANOVA for each group: ldhL cyb2Δ Day 3: 1.43071E-07, ldhL cyb2Δ Day 5: 5.72955E-07, ldhL cyb2Δ Day 8: 0.026939831, ΔRuBisCO Day 3: 0.035678329, ΔRuBisCO Day 3: 0.727779045.

## References


1. T. Gassler, M. Baumschabl, J. Sallaberger, M. Egermeier, D. Mattanovich, Adaptive laboratory evolution and reverse engineering enhances autotrophic growth in *Pichia pastoris*. *Metab. Eng.* **69**, 112–121 (2022).

2. J. D. Young, INCA: A computational platform for isotopically non-stationary metabolic flux analysis. *Bioinformatics* **30**, 1333–1335 (2014).

3. T. Mairinger, *et al.*, Gas chromatography-qadrupole time-of-flight mass spectrometry-based determination of isotopologue and tandem mass isotopomer fractions of primary metabolites for $^{13}$C-metabolic flux analysis. *Anal. Chem.* **87**, 11792–11802 (2015).
